# Comparative Outcomes of Hilar Control in Laparoscopic Partial Nephrectomy: Satinsky Clamp Versus Bulldog Clamp

**DOI:** 10.7759/cureus.95610

**Published:** 2025-10-28

**Authors:** Abdul R Khawaja, Younis Mushtaq, Aamir Mushtaq, Sajad A Para, Sajad A Malik, Saqib Mehdi, Faheem U Islam, Akil L Lone, Arif Hamid, Saleem Wani

**Affiliations:** 1 Urology, Sher-i-Kashmir Institute of Medical Sciences, Srinagar, IND

**Keywords:** bulldog clamps, laparoscopic partial nephrectomy, laparoscopic satinsky clamp, nephron-sparing surgery, small renal mass

## Abstract

Background

Laparoscopic partial nephrectomy (LPN) is a minimally invasive surgery for treating small renal masses using clamps such as Satinsky clamps, bulldog clamps, and vessel loops to control the renal artery and/or vein, reducing blood loss and improving visibility. This study aimed to compare the perioperative outcomes, including operative time, warm ischemia time, and postoperative recovery, of laparoscopic partial nephrectomy performed using either Satinsky or bulldog clamps for hilar control.

Methodology

Data from patients who underwent LPN between 2016 and 2022 were collected retrospectively. Hilar control was achieved using either a Satinsky or Bulldog clamp. Operative time, warm ischemia time, number of ports used, and hospital stay duration were compared. Statistical analysis was performed using R version 4.3.1.

Results

The study included 54 patients who underwent LPN, with a mean age of 57 years. Overall, 61% of the patients were male. Tumors were incidentally detected in 54% and located on the right side in 54%. A laparoscopic Satinsky clamp was used in 31 patients, while a bulldog clamp was used in 23. No significant differences were found between the two groups regarding age, gender, tumor characteristics, T-stage, or RENAL Nephrometry Score (p > 0.05). Warm ischemia time and mean operative time were higher in the bulldog group, but not significantly (p > 0.05). Postoperative stay duration was similar (p = 0.70). The Satinsky group required one additional port.

Conclusions

Both Satinsky and bulldog clamps offer excellent hilar control during LPN with no statistically significant increase in warm ischemia and operative times. The Satinsky clamp required one additional port, which can be used for specimen retrieval. The choice between clamps is influenced by the surgeon’s experience and preference.

## Introduction

Laparoscopic partial nephrectomy (LPN) is a minimally invasive procedure for the management of small renal masses (SRMs), which typically are defined as tumors up to 4 cm in size (T1a) [1]. Indications of the procedure are expanding to include larger (T1b) and more complex masses [2,3]. Extensive literature supports that partial nephrectomy provides comparable oncologic and superior functional outcomes to radical nephrectomy in appropriately selected patients [4,5]. The primary goal of the surgery is to remove the tumor while preserving as much normal kidney tissue as possible, which is critical for maintaining renal function, especially in patients with solitary kidneys or pre-existing renal impairment.

One of the major surgical steps of LPN is achieving proper vascular control. This is typically done by temporarily occluding the blood supply to the kidney through clamping the renal artery and/or vein. This temporary interruption of the blood supply reduces blood loss and allows for a clearer operative field. The duration of ischemia is crucial, as extended blood supply interruption can lead to cellular hypoxia and ischemia-reperfusion injury, which may compromise long-term kidney function [6,7]. Thus, selecting the right clamping technique is vital to reducing ischemia time and promoting a successful outcome.

A variety of methods, including laparoscopic Satinsky clamps, bulldog clamps, and vessel loops, have been used to achieve vascular control during LPN [8,9]. While bulldog clamps require dissection and clamping of individual renal vessels supplying the kidney, a Satinsky clamp may allow en bloc clamping of the vascular pedicle [8].

No previous studies have directly compared the laparoscopic Satinsky clamp against the bulldog clamp in the LPN setting. The rationale for the study is to clarify the clinical implications of clamp selection. The choice of clamp may influence surgical efficiency, technical complexity, and postoperative recovery.

## Materials and methods

We conducted a retrospective, observational study in the Department of Urology at the Sher-i-Kashmir Institute of Sciences (SKIMS), Srinagar, Jammu and Kashmir, India. Data on patients who underwent LPN for SRMs between June 2016 and October 2022 were retrospectively collected from the Department of Medical Records at SKIMS. Patient records wherein the type of clamp used was not entered were excluded from the study. We compared patient demographics including age; gender and presentation; tumor characteristics including laterality, tumor size, tumor location, T-stage; radius, exophytic/endophytic, nearness to the renal sinus or collecting system, anterior or posterior descriptor, and location relative to the polar line (RENAL Nephrometry Score); and perioperative outcomes, including warm ischemia time, operative time, number of ports required, and duration of hospital stay. Intra and postoperative complications and surgical margins were also noted.

LPN was performed through a transperitoneal approach. After positioning in a modified flank position, pneumoperitoneum was created using a Veress needle. The laparoscope was placed through a 12 mm port close to the umbilicus, and two working ports were placed around it. In cases where a Satinsky clamp was used, an additional port was placed away from the working arc of the instruments, preferably in the lower abdominal quadrant. An additional port was used at times for retraction of an enlarged liver or spleen. Hilar vessels were dissected, and the kidney was mobilized. Renal mass was scored circumferentially. The renal pedicle or artery was clamped with either a Satinsky clamp en bloc or a bulldog clamp, respectively, based on the surgeon’s preference. The tumor was excised. Renorrhaphy was performed, and the renal vessels were unclamped. After confirming hemostasis, the tumor was placed into a retrieval bag and removed via a small transverse lower abdominal incision. If a Satinsky clamp was used, the trocar site was incorporated into the wound for specimen removal.

All data were entered in an MS Excel spreadsheet (Microsoft Corp., Redmond, WA, USA), and data analysis was performed using R version 4.3.1. Categorical values were compared using a chi-squared test and are presented as numbers and percentages. Continuous variables were compared using Student’s t-test and the Mann-Whitney test and are presented as means and standard deviation. All results are discussed at a 5% level of significance.

## Results

In total, 60 patients underwent LPN between June 2016 and October 2022. Of these, data on the type of clamp used were not available in six cases. The remaining 54 cases were included in the study. The mean age of the patients was 57 years. Overall, 61% of the patients were males, 54% had their tumors detected incidentally, and 54% had tumors on the right side. The mean warm ischemia time and mean operating time were 27.5 and 187 minutes, respectively. Patients were discharged after a mean duration of three days. A Satinsky clamp was used in 31 cases, and a bulldog clamp was used in 23 cases. Demographic and tumor characteristics of the two groups are shown in Table 1. There was no statistically significant difference between the two groups in terms of demographic characteristics, including age, gender distribution, mode of presentation, and tumor characteristics, including laterality, location, size, T-stage, and RENAL Nephrometry Score.

**Table 1:** Patient demographics and preoperative characteristics. RENAL = R: radius, E: exophytic/endophytic, N: nearness to the renal sinus or collecting system, A: anterior (a) or posterior (p) descriptor, L: location relative to the polar line

Variable	Satinsky group (n = 31)	Bull dog group (n = 23)	Test statistic	P-value
Male sex, number (percentage)	19 (57.6)	14 (60.9)	Χ²	0.975
Age in years, mean ± SD	57.87 ± 11.60	56.04 ± 9.56	t-test	0.5407
Presentation, number (percentage)			Χ²	1
Incidental	17 (54.8)	12 (52.2)		
Flank pain	7 (22.6)	5 (21.7)		
Hematuria	7 (22.6)	6 (26.1)		
Right-sided tumors, number (percentage)	17 (54.8)	12 (52.2)	Χ²	1
Tumor location, number (percentage)			Χ²	1
Lower pole	17 (54.8)	12 (52.2)		
Upper pole	6 (19.4)	5 (21.7)		
Interpolar	8 (25.8)	6(26.1)		
RENAL score, mean ± SD	5.87 ± 1.36	5.30 ± 1.18	U	0.1077
Maximum tumor diameter in mm, mean ± SD	36.61 ± 8.30	34.87 ± 6.82	t-test	0.4148
T-stage, number (percentage)			Χ²	0.7576
T1a	23 (74.2)	18 (78.3)		
T1b	8 (25.8)	5 (21.7)		
Multiple renal arteries, number (percentage)	9 (71)	6 (73.9)	Χ²	0.8112

Perioperative outcomes of the patients are shown in Table 2. Patients in the bulldog group had longer warm ischemia time and operating time, but this difference was not statistically significant (p > 0.05). The number of ports used in the Satinsky group was, on average, one more than that in the bulldog group, and this difference was statistically significant (p < 0.05). There was no significant difference in the duration of hospital stays between the two groups. Three patients in each group required conversion to open due to technical difficulties in suturing. No patient developed any major intraoperative complication. All patients had negative surgical margins on pathology.

**Table 2:** Perioperative outcomes of patients.

Variable	Satinsky group (n = 31)	Bulldog group (n = 23)	Test statistic	P-value
Warm ischemia time in minutes, mean ± SD	22.23 ± 4.31	23.61 ± 3.66	U	0.1935
Operating time in minutes, mean ± SD	170.84 ± 12.21	177.04 ± 13.12	U	0.1107
Number of ports used, mean ± SD	4.29 ± 0.53	3.30 ± 0.47	U	<0.05
Duration of hospital stay in days, mean ± SD	3.39 ± 0.67	3.43 ± 0.66	U	0.7077
Conversion to open, number (percentage)	3 (9.7)	3 (13)	Χ²	0.6971

## Discussion

LPN is a minimally invasive approach for the management of renal masses [3]. Overall, 76% of patients in our study had T1a tumors, while the rest had T1b tumors. Minimally invasive partial nephrectomy, including LPN, is a feasible approach in such patients if it does not compromise oncological or functional outcome [3]. Hilar clamping during LPN prevents bleeding from the cut margin of the kidney and improves vision, albeit at the cost of warm ischemia. The mean warm ischemia time in our patients was 27.5 minutes. Gill et al. showed a similar warm ischemia time when comparing open and laparoscopic partial nephrectomy [10]. Hilar control can be achieved using several techniques, including the application of bulldog clamps, Satinsky clamps, or vessel loops [8,9]. Bulldog clamps require dissection of the individual renal artery and/or vein separately and thus theoretically would require more time. While the Satinsky clamp obviates the need for dissection of individual vessels, allowing en bloc clamping of the renal pedicle, it requires an additional port and may make it difficult to mobilize the kidney. Additionally, it is fraught with the theoretical risk of clashing with the operating surgeon’s working instruments and accidental injury to the renal pedicle [11].

While the two clamps have been compared in the robotic partial nephrectomy setting, to our knowledge, no studies exist comparing the two clamps in the LPN settings [11]. While Abdullah et al. found that the Satinsky group was associated with longer warm ischemia time [11], operating time, and duration of hospital stay, our analysis showed that the bulldog clamp was associated with a statistically insignificant increase in warm ischemia time and operating time compared to the Satinsky clamp. The mean duration of hospital stay does not differ between the two. The oncological outcome, as measured by negative surgical margins, does not differ. The Satinsky clamp needs an extra trocar for placement, but that port can easily be incorporated into the wound for specimen retrieval. In our analysis, neither of the two methods of clamping was associated with any significant intraoperative complication. Given comparable outcomes, clamp choice may depend on individual surgeon familiarity, pending larger trials.

An important caveat relates to the complexity of the tumors in our patients. Overall, 74% of the patients had low-complexity tumors and 26% had intermediate-complexity tumors. No patient had a high complexity renal tumor based on the RENAL Nephrometry Score. Presuming that such high-complexity lesions may require selective clamping of segmental renal vessels, the role of en bloc clamping with Satinsky clamp cannot be adjudged with this study, and such patients may be better dealt with a bulldog clamp.

Our study is limited by its small sample size and retrospective nature. Although no significant statistical differences were found, the small sample size limits the power to detect clinically meaningful effects. Future randomized studies should confirm effects on warm ischemia, complication rate, and renal function.

## Conclusions

While both Satinsky and bulldog clamps provide excellent hilar control during laparoscopic nephron-sparing surgery, the bulldog clamp is associated with a statistically insignificant increase in warm ischemia time and operative time. Although the Satinsky clamp needs one extra port on average, the extra port can easily be incorporated into the wound for specimen retrieval. Surgeon experience and preference influence the use of either of the two clamps.

## References

[1] Richard PO, Violette PD, Bhindi B (2022). Canadian Urological Association guideline: management of small renal masses - full-text. Can Urol Assoc J.

[2] Chenam A, Lau C (2018). Management of small renal masses. Cancer Treat Res.

[3] Ljungberg B, Albiges L, Abu-Ghanem Y (2022). European Association of Urology Guidelines on Renal Cell Carcinoma: the 2022 update. Eur Urol.

[4] Russo P, Huang W (2008). The medical and oncological rationale for partial nephrectomy for the treatment of T1 renal cortical tumors. Urol Clin North Am.

[5] Pierorazio PM, Johnson MH, Patel HD (2016). Management of renal masses and localized renal cancer: systematic review and meta-analysis. J Urol.

[6] Volpe A, Blute ML, Ficarra V (2015). Renal ischemia and function after partial nephrectomy: a collaborative review of the literature. Eur Urol.

[7] Lifshitz DA, Shikanov S, Jeldres C (2009). Laparoscopic partial nephrectomy: predictors of prolonged warm ischemia. J Urol.

[8] Gill IS, Desai MM, Kaouk JH, Meraney AM, Murphy DP, Sung GT, Novick AC (2002). Laparoscopic partial nephrectomy for renal tumor: duplicating open surgical techniques. J Urol.

[9] Lee YH, Kwon JB, Cho SR, Kim JS (2011). A feasible technique for transient vascular occlusion by using a vessel loop and Hem-o-Lok clips in laparoscopic partial nephrectomy. Korean J Urol.

[10] Gill IS, Kavoussi LR, Lane BR (2007). Comparison of 1,800 laparoscopic and open partial nephrectomies for single renal tumors. J Urol.

[11] Abdullah N, Rahbar H, Barod R (2017). Use of the Satinsky clamp for hilar clamping during robotic partial nephrectomy: indications, technique, and multi-center outcomes. J Robot Surg.

